# METTL3 promotes adriamycin resistance in MCF-7 breast cancer cells by accelerating pri-microRNA-221-3p maturation in a m6A-dependent manner

**DOI:** 10.1038/s12276-020-00510-w

**Published:** 2021-01-08

**Authors:** Xiaoping Pan, Xiaolv Hong, Sumei Li, Ping Meng, Feng Xiao

**Affiliations:** 1 Clinical Laboratory, Huadu Hospital, Southern Medical University, 510800 Guangzhou, P. R. China; 2 Department of Infectious Disease, Huadu Hospital, Southern Medical University, 510800 Guangzhou, P. R. China; 3 Central Laboratory, Huadu Hospital, Southern Medical University, 510800 Guangzhou, P. R. China

**Keywords:** Breast cancer, Cell biology

## Abstract

Breast cancer (BC) is the most prevalent malignant neoplasm among women and is the fifth most common cause of cancer-associated death worldwide. Acquired chemoresistance driven by genetic and epigenetic alterations is a significant clinical challenge in treating BC. However, the mechanism of BC cell resistance to adriamycin (ADR) remains to be elucidated. In this study, we identified the methyltransferase-like 3/microRNA-221-3p/homeodomain-interacting protein kinase 2/Che-1 (METTL3/miR-221-3p/HIPK2/Che-1) axis as a novel signaling event that may be responsible for resistance of BC cells to ADR. A dual-luciferase reporter gene assay was employed to test the presence of miR-221-3p binding sites in the 3′UTR of HIPK2. Drug resistance was evaluated by immunoblotting multidrug resistance protein 1 (MDR1) and breast cancer resistance protein (BCRP). Cultured ADR-resistant MCF-7 cells were assayed for their half maximal inhibitory concentration (IC50) values and apoptosis using an MTT assay and Annexin V-FITC/PI-labeled flow cytometry, and the cells were then xenografted into nude mice. METTL3 knockdown was shown to reduce the expression of miR-221-3p by reducing pri-miR-221-3p m6A mRNA methylation, thereby reducing the IC50 value of ADR-resistant MCF-7 cells, reducing the expression of MDR1 and BCRP, and inducing apoptosis. Mechanistically, miR-221-3p was demonstrated to negatively regulate HIPK2 and upregulate its direct target Che-1, thus leading to enhanced drug resistance in ADR-resistant MCF-7 cells. In vitro results were reproduced in nude mice xenografted with ADR-resistant MCF-7 cells. Our work elucidates an epigenetic mechanism of acquired chemoresistance in BC, in support of the METTL3/miR-221-3p/HIPK2/Che-1 axis as a therapeutic target for the improvement of chemotherapy.

## Breast cancer: Enzyme drives resistance to chemotherapy

An enzyme involved in promoting drug resistance in breast cancer could be targeted to improve the success of chemotherapy. Abnormal expression of microRNAs is well documented in cancers, and has been implicated in tumorigenesis, chemoresistance and metastasis. These abnormalities may be linked to an enzyme called methyltransferase-like 3 (METTL3), which is known to promote chemoresistance in lung and bladder cancers. Xiaoping Pan at the Southern Medical University in Guangzhou, China, and co-workers examined the role of METTL3 in conferring resistance to the drug adriamycin in breast cancer cells. The team screened for differences in microRNA expression between breast cancer and healthy cells. They found that one microRNA, miR-221-3p, was overexpressed in chemoresistant breast cancer cells, via a signaling pathway involving METTL3. The results highlight the potential for therapeutic strategies linked to METTL3.

## Introduction

Breast cancer (BC) is the most prevalently diagnosed malignancy in the female population, with 2,088,849 new cases diagnosed and 626,679 deaths reported in 2018 worldwide, accounting for 11.6% and 6.6% of cases and deaths associated with cancer, respectively^1^. Adriamycin (ADR), also known as doxorubicin, is a member of the anthracycline drug family clinically applied for the treatment of multiple types of cancers, including BC, yet resistance of cancer cells and the effects of ADR on healthy tissues remain challenges to achieving satisfactory therapeutic effects^2,3^. Therefore, novel treatment modalities to alleviate BC cell resistance to ADR are expected to improve the therapeutic effects.

N6-methyladenosine (m6A) modification has been found to play a part in regulating cellular biological processes^4^. Additionally, microRNAs (miRNAs) emerge as potential noninvasive cancer biomarkers implicated in tumorigenesis as well as drug resistance and metastasis considering their aberrant expression profile^5^. Methyltransferase-like 3 (METTL3)-induced m6A messenger RNA (mRNA) methylation has been documented to mediate drug resistance to cisplatin in nonsmall cell lung cancer, the underlying regulatory mechanism of which involves miR-1914-3p^6^. In the context of bladder cancer, METTL3 has been indicated to promote the expression of miR-221 by contributing to m6A modification through modulation of the pri-miR-221/222 process^7^. More importantly, METTL3 facilitates the tumorigenesis of BC^8^. In silico analysis prior to our investigation showed the upregulation and significance of miR-221-3p in BC. Concordantly, available evidence has revealed significantly higher expression of miR-221 in the plasma of patients with BC, serving as an independent biomarker to predict chemotherapeutic response^9^. Additionally, homeodomain-interacting protein kinases (HIPKs) have been recognized for their regulatory potential from the perspectives of apoptosis, response to DNA damage, cellular proliferation and extracellular stimuli management through their interplay with miRNAs^10^. Notably, the bioinformatics analysis in our study further revealed HIPK2 as a putative target gene of miR-221-3p. Furthermore, HIPK2 has been found to downregulate the expression of Che-1/AATF (Che-1), a regulator of gene transcription, cellular proliferative potential and resistance to anticancer drugs^11,12^. In addition, multidrug resistance gene 1 (MDR1) and breast cancer resistance protein (BCRP) have been suggested to be of great importance in mediating the drug resistance of patients with BC^13^. Given the aforementioned review, we sought to investigate the communication of METTL3 with BC cell resistance to ADR, involving its crosstalk with the functional axis of miR-221-3p/HIPK2/Che-1 as well as its regulation of the expression of MDR1 and BCRP.

## Materials and methods

### Cell lines and transfection

Human breast epithelial MCF-10A cells, ADR-resistant MCF-7 (MCF-7/ADR) cells and ADR-sensitive MCF-7 (MCF-7/S) cells were purchased from the Cell Bank of Typical Culture Preservation Committee of Chinese Academy of Science (Shanghai, China). MCF-10A and MCF-7/S were maintained in Roswell Park Memorial Institute-1640 (RPMI-1640) medium (Gibco by Life Technologies, Grand Island, NY, USA) and MCF-7/ADR in 1.0 µmol/L ADR-supplemented RPMI-1640 with exposure to an atmosphere of 5% CO_2_ at 37 °C. METTL3-specific short hairpin RNA (shRNA); full-length expression plasmids for METTL3, HIPK2, and Che-1; and miR-221-3p-specific miRNA mimic and inhibitor (all purchased from GenePharma, Shanghai, China) were introduced into MCF-7/ADR cells by using Lipofectamine 2000 reagent (11668019, Invitrogen, Carlsbad, CA, USA) according to the manufacturer’s instructions.

### Reverse transcription quantitative polymerase chain reaction (RT-qPCR)

Total RNA was extracted using TRIzol reagent (Invitrogen, Carlsbad, CA, USA), followed by RNA quality control by nanodrop2000 (1011U, Nanodrop, USA). cDNA was generated using the TaqMan MicroRNA Assays RT primer (4427975, Applied Biosystems, Foster City, CA, USA)/PrimeScript RT reagent Kit (RR047A, Takara, Shiga, Japan) following the instructions provided by the manufacturer (Takara, Dalian, Liaoning, China). Quantification of miR-221-3p expression was checked by the CFX96 System (BioRad, USA), with each run performed in triplicate. The expression of miR-221-3p was normalized to the expression of U6 and calculated by using the 2^−△△CT^ method. Primer sequences were as follows: miR-221-3p (forward): 5′-CCCAGCATTTCTGACTGTTG-3′; miR-221-3p (reverse): 5′-AACGCTTCACGAATTTGCGT-3′; U6 (forward): 5′-GCTTCGGCAGCACATATACTAAAAT-3′; U6 (reverse): 5′-CGCTTCACGAATTTGCGTGTCAT-3′.

### Immunoblotting

Cell lysate was made by supplementing ice-cold radioimmunoprecipitation buffer (P0013C, Beyotime, Shanghai, China) with protease inhibitors. Protein extractions (50 μg for each run) were performed on 10% sodium dodecyl sulfate polyacrylamide gel electrophoresis (SDS-PAGE) and then wet-transferred onto the membrane (polyvinylidene fluoride). Immunoblots were allowed to react with rabbit primary antibodies (all purchased from Abcam, Cambridge, UK) to METTL3 (1:1000; ab195352), HIPK2 (1:500; ab28507), MDR1 (1:1000; ab129450), BCRP (1:1000; ab207732), B-cell lymphoma-2 (Bcl-2) associated protein X (Bax) (1:2000; ab182733), Bcl-2 (1:1000; ab32124), and glyceraldehyde-3-phosphate dehydrogenase (GAPDH) (1:2500; ab9485) and were visualized by using horseradish peroxidase-labeled goat antirabbit immunoglobulin G (IgG) (1:2000; ab97051) and enhanced chemiluminescence (ECL) reagents (BB-3501, Amersham Pharmacia, UK). Each sample was run in triplicate. Gray values of immunoblots were normalized to GAPDH and analyzed using ImageJ v1.48u software (National Institutes of Health, Bethesda, Maryland, USA).

### Luciferase reporter assay

The pmirGLO-based luciferase reporter plasmids (Promega, Madison, WI, USA) containing wild-type HIPK2 (termed HIPK2-Wt) or HIPK2 mutated at the putative miR-221-3p binding sites (termed HIPK2-Mut) were designed. The desired pmirGLO-HIPK2-Wt and pmirGLO-HIPK2-Mut plasmids with either miR-221-3p-specific miRNA mimic or miRNA mimic negative control (NC) were delivered into HEK293T cells by liposome-mediated transfection methods. Forty-eight hours later, a portion of the HEK293T cell lysate (100 μL) was added to an equal amount of Renilla luciferase, and the luminescence was determined by a microplate reader (SpectraMax M5, Molecular Devices, USA). Another part of the HEK293T cell lysate (100 μL) was added to an equal amount of firefly luciferase, and the luminescence was determined by a SpectraMax M5 microplate reader. The luminescence of firefly luciferase was analyzed relative to that of Renilla luciferase.

### m6A RNA immunoprecipitation (RIP) assay

Immunoprecipitation was performed using anti-DGCR 8 antibody (1:100, ab191875, Abcam) and anti-m6A antibody (1:500, ab151230, Abcam) relative to normal IgG (1:500, ab109489, Abcam) previously conjugated to protein A/G magnetic beads (Life Technologies, USA) in immunoprecipitation buffer (20 mM Tris pH 7.5, 140 mM NaCl, 1% Nonidet P-40, 2 mM ethylenediamine tetraacetic acid) and incubated with proteinase K. Immunoprecipitated RNA was extracted by a standard phenol/chloroform procedure and analyzed by RT-qPCR analysis using primers for pri-miRNAs, normalizing to the input.

### Cell survival assay

MCF-7/ADR cells were seeded into 96-well plates with 8 × 10^3^ cells per well for three parallel experiments. Twenty-four hours later, MCF-7/ADR cells were exposed to ADR at increasing concentrations (0, 20, 40, 60, 80 and 100 μmol/L) for 48 h. ADR-treated cells were incubated with 10 μL 3-(4,5)-dimethylthiazol (-z-y1)-3,5-diphenyltetrazolium bromide (MTT) solution (5 mg/mL, Sigma-Aldrich, St Louis, MO, USA) for 4 h. Two hours later, the media was renewed using 150 μL dimethyl sulfoxide (DMSO, Sigma-Aldrich) in each well to dissolve the formazan crystals. Absorbance was read at 570 nm using a microplate reader (Bio-Rad, Hercules, CA, USA) and was used to draw growth curves.

### Cell apoptosis assays

An apoptosis assay was performed using the fluorescein isothiocyanate (FITC) Annexin V Apoptosis Detection Kit (K201-100, Biovision, USA) following the protocol. Propidium iodide (PI) was used in conjunction with Annexin V to determine if cells were viable, apoptotic, or necrotic, and cells were then analyzed by a flow cytometer (FACScan®; BD Biosciences, San Jose, CA, USA) equipped with CellQuest software (BD Biosciences).

### Xenografts of human BC cells in nude mice

A total of 24 BALB/c mice (aged 4–6 weeks; weighing 17 to 22 g), purchased from Hunan SJA Laboratory Animal Co., Ltd. (Changsha, Hunan, China), were kept under specific pathogen-free conditions. Cell suspensions (2 × 10^6^ cells/mL) made with MCF-7/ADR cells stably expressing METTL3 and/or miR-221-3p inhibitor were subcutaneously implanted into each mouse. One week later, xenografted mice were injected with 0.1 mL ADR (25 mg/kg, intraperitoneal injection) twice a week. The growth of human BC xenografts in mice was monitored every week. Six weeks after ADR injection, the mice were euthanized by cervical dislocation. Animals were cared for in accordance with the Guide for the Care and Use of Laboratory Animals and approved by the Laboratory Animal Care and Use Committee of Huadu Hospital, Southern Medical University.

### Analysis of statistical significance

Measurement data are shown as the mean ± standard deviation of at least three independent experiments performed in triplicate. Statistical comparisons were performed using Student’s *t* test when only two groups were compared or by Tukey’s test-corrected one-way analysis of variance (ANOVA) when more than two groups were compared. Variables were analyzed at different time points using Bonferroni-corrected repeated measures ANOVA. All statistical analyses were completed with SPSS 21.0 software (IBM, Armonk, NY, USA), with two-tailed *p* < 0.05 as a level of statistical significance.

## Results

### Upregulated miR-221-3p was associated with ADR resistance in BC tissue

**Fig. 1:**
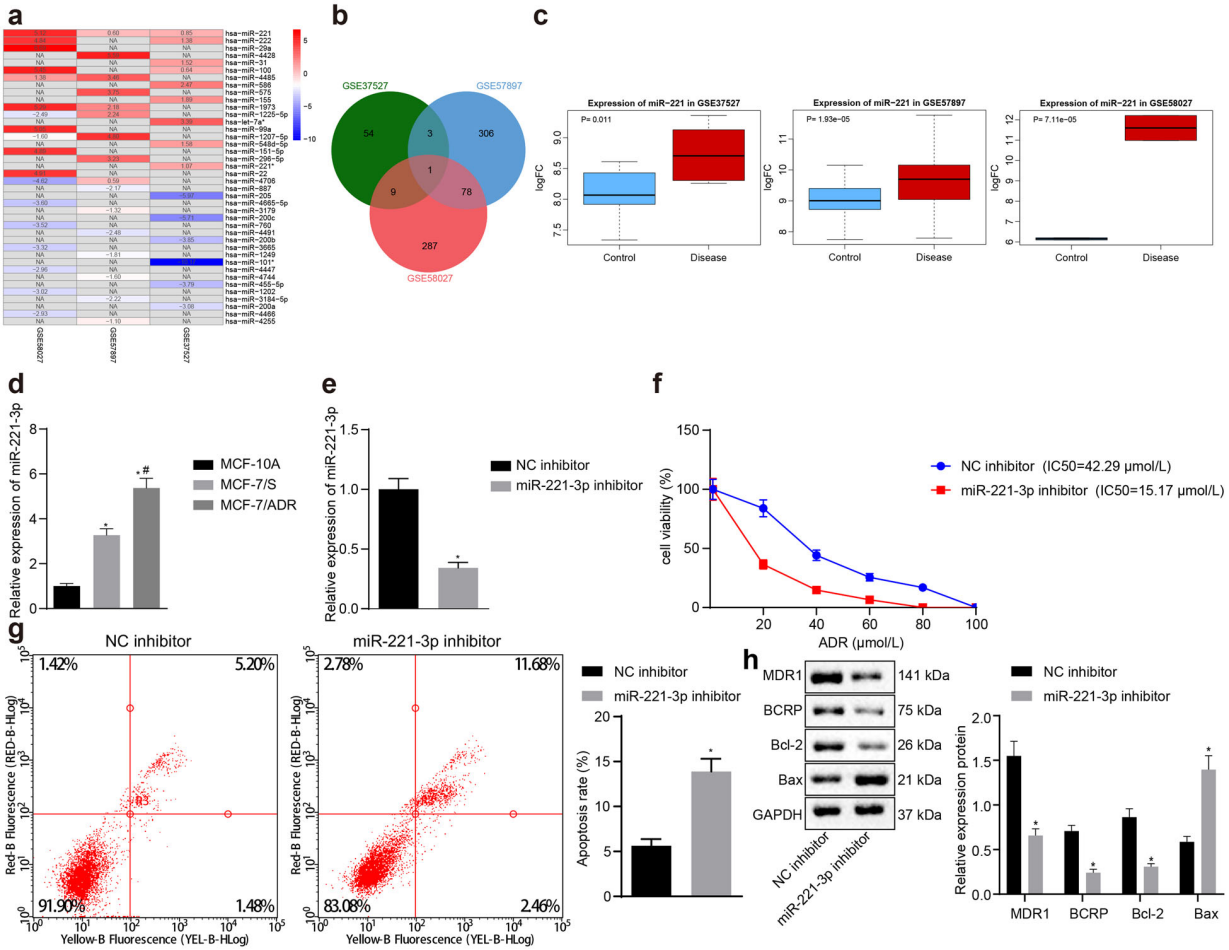
Upregulated miR-221-3p is associated with ADR resistance in BC. **a** Heatmap showing differentially expressed miRNAs between BC and normal breast tissue samples in the GSE37527 (67 miRNAs, 6 tumor samples, and 6 normal samples), GSE57897 (388 miRNAs, 422 tumor samples, and 31 normal samples) and GSE58027 (375 miRNAs, 2 tumor samples, and 2 normal samples) datasets with |logFoldChange|>0.5 deposited in the GEO (http://www.ncbi.nlm.nih.gov/geo/). **b** Venn diagram showing miR-221-3p as the common differentially expressed miRNA between BC and normal breast tissue samples. **c** Expression box of miR-221-3p by the GSE37527 (left), GSE58027 (right) and GSE57897 (middle) datasets. **d** The expression of miR-221-3p was determined by RT-qPCR among MCF-10A human breast epithelial cells, MCF-7/ADR cells and MCF-7/S cells normalized to U6 expression; *p < 0.05 compared to MCF-10A and #*p* < 0.05 compared to MCF-7/S cells by Tukey’s test-corrected one-way ANOVA. **e** Inhibition of miR-221-3p by miR-221-3p inhibitor. **f** The IC50 of miR-221-3p inhibitor-treated MCF-7/ADR cells was examined by MTT assays. **g** The apoptosis of miR-221-3p inhibitor-treated MCF-7/ADR cells was examined by Annexin V-FITC/PI-labeled flow cytometry. **h** Immunoblots and quantification of BCRP, MDR1, Bcl-2 and Bax proteins in miR-221-3p inhibitor-treated MCF-7/ADR cells, normalized to GAPDH. *p < 0.05 compared to NC inhibitor by unpaired *t*-test for **e**, **g**, and **h**, by Bonferroni-corrected repeated measures ANOVA for **f**.

Differentially expressed miRNAs (Fig. 1a) between BC and normal breast tissue samples were screened by analyzing miRNA microarray data in the GSE37527, GSE57897 and GSE58027 datasets of the Gene Expression Omnibus (GEO) database, and miR-221-3p was identified by Venn diagram (Fig. 1b) as a common differentially expressed miRNA. miR-221-3p was upregulated in the microarray datasets GSE37527, GSE58027, and GSE57897 (Fig. 1c). To validate the association between the expression pattern of miR-221-3p and BC chemoresistance, we determined the expression of miR-221-3p among MCF-10A human breast epithelial cells, MCF-7/ADR cells and MCF-7/S cells by RT-qPCR. As expected, miR-221-3p exhibited a higher expression level in BC cells than in MCF-10A cells and in MCF-7/ADR cells than in MCF-7/S cells (Fig. 1d). Subsequently, we attempted to examine the effects of miR-221-3p on BC chemoresistance. To do so, we successfully introduced a miR-221-3p-specific inhibitor into MCF-7/ADR cells (Fig. 1e). miR-221-3p inhibitor-treated MCF-7/ADR cells were subjected to MTT assays and Annexin V-FITC/PI-labeled flow cytometry. As expected, inhibition of miR-221-3p reduced the IC50 of MCF-7/ADR cells but enhanced the apoptosis of MCF-7/ADR cells (Fig. 1f, g). BCRP and MDR1 are two MDR proteins that are associated with clinical resistance to chemotherapy in BC patients. Immunoblotting analysis of BCRP, MDR1, apoptosis-related proteins Bcl-2 and Bax was performed in miR-221-3p inhibitor-treated MCF-7/ADR cells. We found that inhibition of miR-221-3p diminished the protein expression of BCRP, MDR1 and Bcl-2 but increased the protein expression of Bax (Fig. 1h). Overall, these data suggest that overexpression of miR-221-3p may be associated with ADR resistance in BC.

### METTL3 increased the expression of miR-221-3p by enhancing pri-miR-221-3p m6A mRNA methylation in MCF-7/ADR cells

**Fig. 2:**
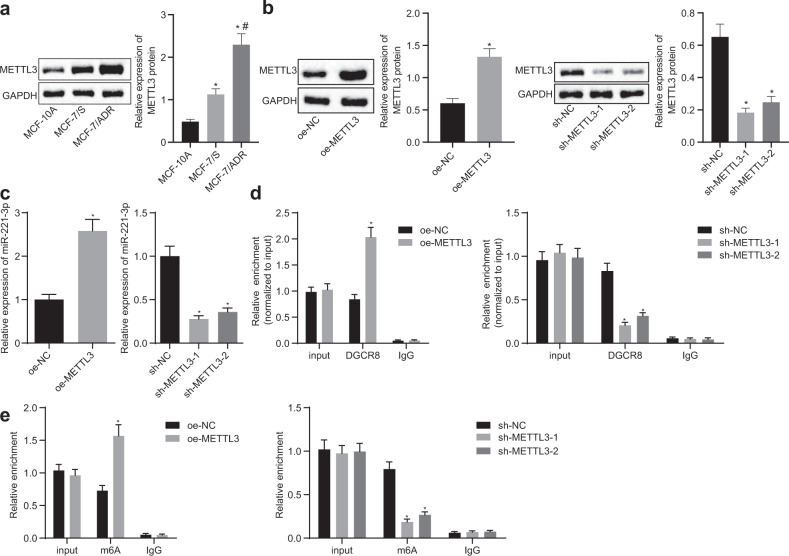
METTL3 increases the expression of miR-221-3p by enhancing pri-miR-221-3p m6A mRNA methylation in MCF-7/ADR cells. **a**, **b** Immunoblots and quantification of METTL3 among MCF-10A human breast epithelial cells, MCF-7/ADR cells, MCF-7/S cells, METTL3 overexpression and knockdown MCF-7/ADR cells normalized to GAPDH. **c** The expression of miR-221-3p was determined by RT-qPCR in METTL3 overexpression and knockdown MCF-7/ADR cells normalized to U6 expression. **d**, **e** Me-RIP assay was performed using anti-DGCR8 and anti-m6A in METTL3 overexpression and knockdown MCF-7/ADR cells, and the immunoprecipitated RNA was subjected to RT-qPCR of pri-miR-221-3p. Statistical comparisons were performed using unpaired *t*-test when only two groups were compared or by Tukey’s test-corrected ANOVA when more than two groups were compared. *p < 0.05 compared to MCF-10A, empty vector (oe-NC), or scramble shRNA (sh-NC) and #*p* < 0.05 com*p*ared to MCF-7/S cells.

Our first demonstration that miR-221-3p expression contributes to the clinical resistance of BC to chemotherapy revealed the need for further investigation of the mechanism of miR-221-3p in BC chemoresistance. METTL3 could promote m6A mRNA methylation of pri-miRNA in human cancers based on previous evidence. Thus, we propose a hypothesis that METTL3 mediates pri-miR-221-3p maturation by accelerating m6A mRNA methylation. To test this hypothesis, our first step is to immunoblot METTL3 among MCF-10A human breast epithelial cells, MCF-7/ADR cells and MCF-7/S cells. METTL3 exhibited a higher expression level in BC cells than in MCF-10A cells and in MCF-7/ADR cells than in MCF-7/S cells (Fig. 2a). The subsequent experiment focused on characterizing the functional role of METTL3 in BC chemoresistance. We successfully constructed METTL3 overexpression and knockdown MCF-7/ADR cells, which were confirmed by RT-qPCR and immunoblotting (Fig. 2b, c). Likewise, miR-221-3p was upregulated or downregulated in response to METTL3 overexpression or knockdown in MCF-7/ADR cells. To test the epigenetic regulation of METTL3 on pri-miR-221-3p, a Me-RIP assay was performed using anti-DGCR8 and anti-m6A antibodies in MCF-7/ADR cells. We found that more pri-miR-221-3p was immunoprecipitated by anti-DGCR8 and anti-m6A antibodies than by IgG. METTL3 overexpression yielded increased pri-miR-221-3p immunoprecipitation with anti-DGCR8 and anti-m6A antibodies, and loss-of-function analysis verified the findings from a gain-of-function study (Fig. 2d, e). Overall, we conclude that METTL3 overexpression may be associated with drug resistance in BC and that METTL3 increases the expression of miR-221-3p by enhancing pri-miR-221-3p m6A mRNA methylation.

### METTL3-mediated pri-miR-221-3p maturation promoted ADR resistance in MCF-7 cells

**Fig. 3:**
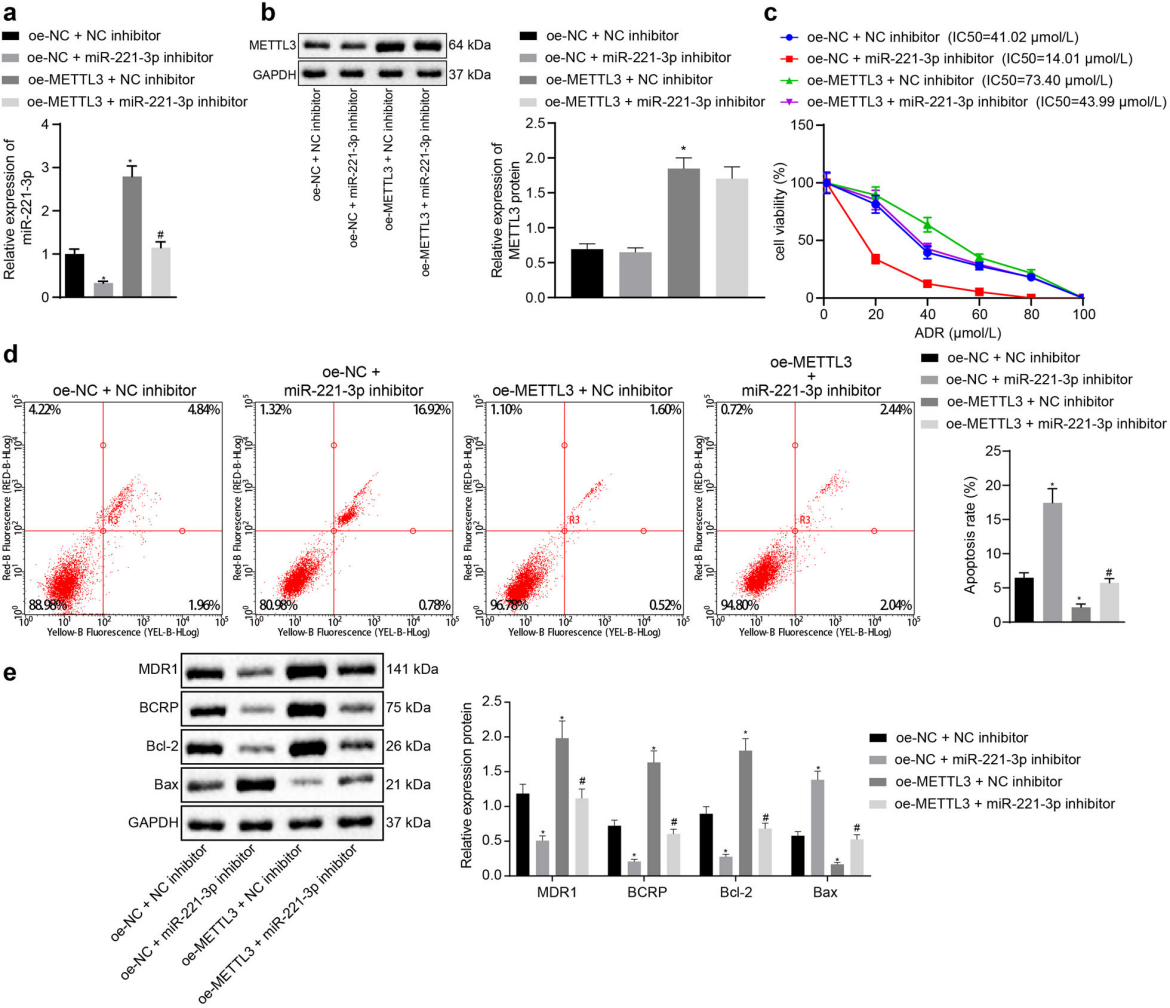
METTL3 modulates ADR resistance by enhancing pri-miR-221-3p maturation. **a** The expression of miR-221-3p was determined by RT-qPCR in MCF-7/ADR cells treated with expression vector containing the METTL3 and/or miR-221-3p-specific inhibitor, normalized to U6 expression. **b** Immunoblots and quantification of METTL3 in MCF-7/ADR cells treated with expression vector containing the METTL3 and/or miR-221-3p-specific inhibitor, normalized to GAPDH. **c** The IC50 of MCF-7/ADR cells treated with an expression vector containing METTL3 and/or a miR-221-3p-specific inhibitor was examined by MTT assays. **d** Apoptosis of MCF-7/ADR cells treated with an expression vector containing METTL3 and/or a miR-221-3p-specific inhibitor was examined by Annexin V-FITC/PI-labeled flow cytometry. **e** Immunoblots and quantification of BCRP, MDR1, Bcl-2, and Bax proteins in MCF-7/ADR cells treated with expression vector containing the METTL3 and/or miR-221-3p-specific inhibitor, normalized to GAPDH. *p < 0.05 compared to empty vector (oe-NC) + NC inhibitor and #*p* < 0.05 compared to expression vector containing the METTL3 + NC inhibitor by Tukey’s test-corrected ANOVA for **a**, **b**, **d**, and **e**, by Bonferroni-corrected repeated measures ANOVA for **c**.

To investigate the action of METTL3 on the drug resistance of BC cells, METTL3 was silenced in MCF-7/ADR cells, followed by evaluation of cell viability and apoptosis results using an MTT assay (Supplementary Fig. 1a) and flow cytometric analysis (Supplementary Fig. 1b), respectively. Cell viability and IC50 values were significantly suppressed, while cell apoptosis was potentiated in response to METTL3 silencing. According to further immunoblotting, the expression levels of MDR1, BCRP, and Bcl-2 were diminished, and the Bax level was elevated (Supplementary Fig. 1c). These findings demonstrated the suppressive action of silencing METTL3 on the drug resistance of BC cells. The purpose of the following experiments was to study whether METTL3 modulates ADR resistance by enhancing pri-miR-221-3p maturation. miR-221-3p inhibition resistance and METTL3 overexpression was performed in MCF-7/ADR cells by using a miR-221-3p-specific inhibitor and expression vector containing METTL3, which was demonstrated by RT-qPCR and immunoblotting (Fig. 3a, b). We then performed MTT assays and Annexin V-FITC/PI-labeled flow cytometry and found that inhibition of miR-221-3p reduced the IC50 of METTL3-overexpressing MCF-7/ADR cells but induced apoptosis (Fig. 3c, d). Immunoblotting analysis found that inhibition of miR-221-3p diminished the protein expression of BCRP, MDR1, and Bcl-2 but increased the protein expression of Bax in METTL3-overexpressing MCF-7/ADR cells (Fig. 3e). The above results confirm the hypothesis that METTL3 modulates ADR resistance by enhancing pri-miR-221-3p maturation.

### miR-221-3p negatively regulated HIPK2 in MCF-7/ADR cells

**Fig. 4:**
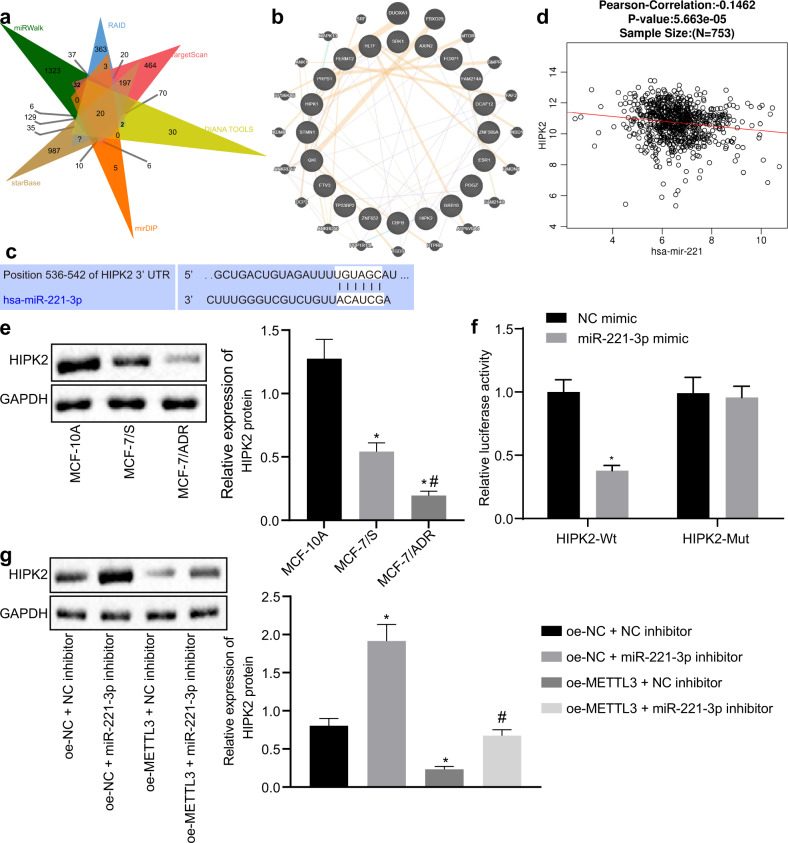
HIPK2 is the target gene of miR-221-3p in MCF-7/ADR cells. **a** Twenty common putative target genes of miR-221-3p among the miRWalk (http://mirwalk.umm.uni-heidelberg.de/), RAID (http://www.rna-society.org/raid2/index.html), TargetScan (http://www.targetscan.org/vert_71/), DIANA TOOLS (http://diana.imis.athena-innovation.gr/DianaTools/), mirDIP (http://ophid.utoronto.ca/mirDIP/) and starBase (http://starbase.sysu.edu.cn/) databases, shown by Venn diagram. **b** Twenty putative target genes of miR-221-3p mapped into the GeneMANIA database (http://genemania.org/), with a constructed PPI network. **c** The miR-221-3p binding sites in the 3′UTR of HIPK2 by TargetScan database analysis. **d** Correlation between miR-221 and HIPK2 in BC predicted by LinkedOmics from TCGA database (Cor = −0.1462, *p* = 5.663E−05). **e** Immunoblots and quantification of HIPK2 among MCF-10A human breast epithelial cells, MCF-7/ADR cells, and MCF-7/S cells. **f** miR-221-3p binding with HIPK2 verified by the luciferase reporter assay. **g** Immunoblots and quantification of HIPK2 in MCF-7/ADR cells treated with expression vector containing the METTL3 and/or miR-221-3p-specific inhibitor, normalized to GAPDH. Statistical comparisons were performed using unpaired *t*-test when only two groups were compared or by Tukey’s test-corrected ANOVA when more than two groups were compared. *p < 0.05 compared to MCF-10A, NC mimic, or empty vector (oe-NC) + NC inhibitor and #*p* < 0.05 com*p*ared to MCF-7/S cells or expression vector containing the METTL3 + NC inhibitor.

To identify the target gene of miR-221-3p in BC, we performed miRNA-mRNA prediction using the miRWalk, RAID, TargetScan, DIANA TOOLS, mirDIP, and starBase databases, and 20 common putative target genes of miR-221-3p were identified by Venn diagram (Fig. 4a). These 20 target genes were mapped into the GeneMANIA database for the PPI network (Fig. 4b), and the HIPK2 gene ranked third (Table 1). QKI has been reported to be targeted by miR-221, which targets QKI to potentiate tumorigenicity of human colorectal cancer stem cells^14^. Despite the implication of HLTF in the development of BC, its association with drug resistance remains unclear^15^. Of note, HIPK2 has been indicated to be involved in the drug resistance of BC cells^16^. Since HIPK2 shares certain functions with miR-221, HIPK2 was selected as a key target gene of miR-221-3p for further investigation in our study. The miR-221-3p binding sites in the 3′untranslated region (3′UTR) of HIPK2 were predicted by TargetScan database analysis (Fig. 4c). Prediction results on LinkedOmics revealed a negative correlation between miR-221 and HIPK2 (Cor = −0.1462, *p* = 5.663E−05, Fig. 4d). HIPK2 showed a lower expression level in BC cells than in MCF-10A cells and in MCF-7/ADR cells than in MCF-7/S cells (Fig. 4e). We also found that the miR-221-3p mimic reduced the luciferase activity in the reporter gene containing the seed sequence in the 3′UTR of HIPK2, compared with the activity in the mutant reporter gene (Fig. 4f). Immunoblotting analysis of HIPK2 showed that METTL3 overexpression diminished the expression of HIPK2 and that miR-221-3p inhibition increased the expression of HIPK2 in MCF-7/ADR cells (Fig. 4g). miR-221-3p inhibition elevated the expression of HIPK2 in METTL3-overexpressing MCF-7/ADR cells.

**Table 1:** PPI network of 20 common putative target genes of miR-221-3p.

Gene	Degree	Sum_weight	Gene	Degree	Sum_weight
QKI	10	0.98	CBFB	3	0.03
HLTF	9	0.71	ESR1	3	0.03
HIPK2	8	0.81	SBK1	3	0.02
HIPK1	8	0.36	PRPS1	2	0.25
FERMT2	6	0.87	GRB10	2	0.04
STMN1	5	0.79	AXIN2	2	0.03
ZNF652	4	0.04	DCAF12	2	0.03
FAM214A	3	1.01	ZNF385A	2	0.02
TP53BP2	3	0.04	FOXP1	0	0.00
POGZ	3	0.03	ETV3	0	0.00

Degree indicates the numbers of interactions of the gene with other genes; Sum_Weight indicates the sum of scores of the interactions of the gene with other genes.

*PPI* protein–protein interaction, *miR* microRNA.

### miR-221-3p promoted ADR resistance by modulating the interaction between HIPK2 and Che-1 in MCF-7 cells

**Fig. 5:**
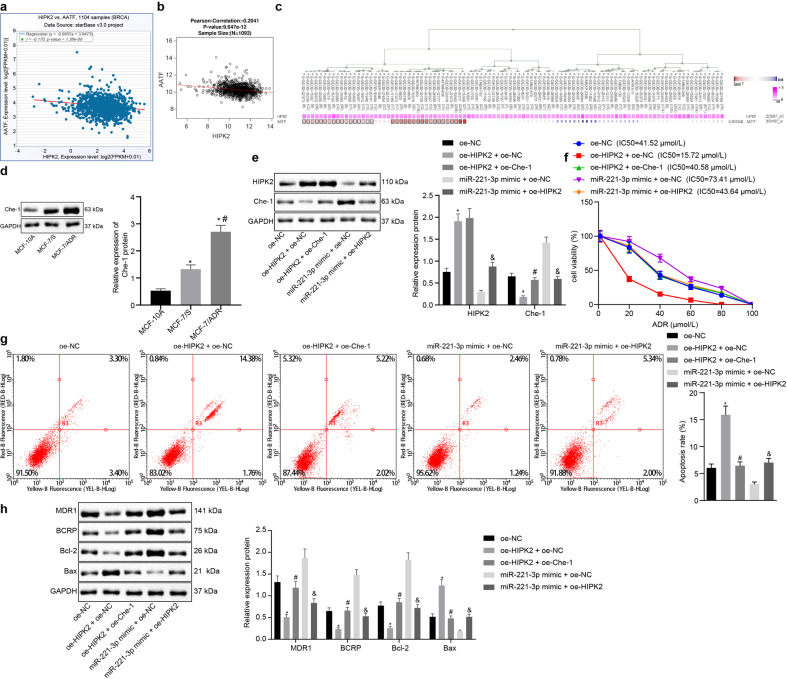
miR-221-3p promotes ADR resistance by modulating HIPK2 interaction with Che-1 in MCF-7 cells. **a** The starBase database analysis of the association of HIPK2 with Che-1. **b** The LinkedOmics database (http://www.linkedomics.org) analysis of the association of HIPK2 with Che-1. **c** HIPK2 coexpressed with Che-1 by MEM database analysis (https://biit.cs.ut.ee/mem/index.cgi). **d** Immunoblots and quantification of Che-1 among MCF-10A human breast epithelial cells, MCF-7/ADR cells, and MCF-7/S cells normalized to GAPDH. **e** Immunoblots and quantification of HIPK2 and Che-1 in MCF-7/ADR cells in response to overexpression of HIPK2, Che-1, or miR-221-3p, normalized to GAPDH. **f** The IC50 of MCF-7/ADR cells in response to overexpression of HIPK2, Che-1, or miR-221-3p, examined by MTT assays. **g** The apoptosis of MCF-7/ADR cells in response to overexpression of HIPK2, Che-1, or miR-221-3p, examined by Annexin V-FITC/PI-labeled flow cytometry. **h** Immunoblots and quantification of BCRP, MDR1, Bcl-2 and Bax proteins in MCF-7/ADR cells in response to overexpression of HIPK2, Che-1, or miR-221-3p normalized to GAPDH. *p < 0.05 compared to MCF-10A and oe-NC, #*p* < 0.05 compared to oe-HIPK2 + oe-NC and MCF-7/S cells, and &*p* < 0.05 compared to miR-221-3p mimic + empty vector (oe-NC) by Tukey’s test-corrected ANOVA or by Bonferroni-corrected repeated measures ANOVA only for **f**.

The starBase database analysis showed that HIPK2 is negatively correlated with Che-1 (named AATF in the NCBI public database) (Fig. 5a), which was further demonstrated by the LinkedOmics analysis (Fig. 5b) and the MEM analysis (Fig. 5c). Therefore, we hypothesize that Che-1 is the direct target of HIPK2 and mediates BC chemoresistance. Immunoblotting analysis revealed that Che-1 showed a higher expression level in BC cells than in MCF-10A cells and in MCF-7/ADR cells than in MCF-7/S cells (Fig. 5d). Then, we manipulated the expression of miR-221-3p, HIPK2, and Che-1 using an expression vector containing HIPK2, Che-1, or miR-221-3p mimic (Fig. 5e). HIPK2 overexpression reduced the expression of Che-1, and Che-1 overexpression did not influence HIPK2 in MCF-7/ADR cells. Elevation of miR-221-3p by its specific mimic increased the expression of Che-1, while HIPK2 overexpression reduced the expression of Che-1 against the miR-221-3p mimic. We then performed MTT assays and Annexin V-FITC/PI-labeled flow cytometry and found that HIPK2 overexpression reduced the IC50 of MCF-7/ADR cells but facilitated apoptosis, while Che-1 overexpression increased the IC50 of HIPK2-overexpressing MCF-7/ADR cells but reduced apoptosis (Fig. 5f, g). We also found that HIPK2 overexpression could reduce the IC50 of miR-221-3p mimic-treated MCF-7/ADR cells but facilitate apoptosis. Immunoblotting analysis revealed that HIPK2 overexpression diminished the protein expression of BCRP, MDR1, and Bcl-2 but increased the protein expression of Bax in MCF-7/ADR cells, which was inhibited by Che-1 overexpression (Fig. 5h). In parallel, HIPK2 overexpression partially negated the regulation of miR-221-3p on BCRP, MDR1, Bcl-2, and Bax in MCF-7/ADR cells. The above results confirm the hypothesis that miR-221-3p promotes ADR resistance by modulating the HIPK2/Che-1 signaling pathway in MCF-7 cells.

### METTL3 promoted BC cell resistance to ADR by enhancing miR-221-3p expression in vivo

**Fig. 6:**
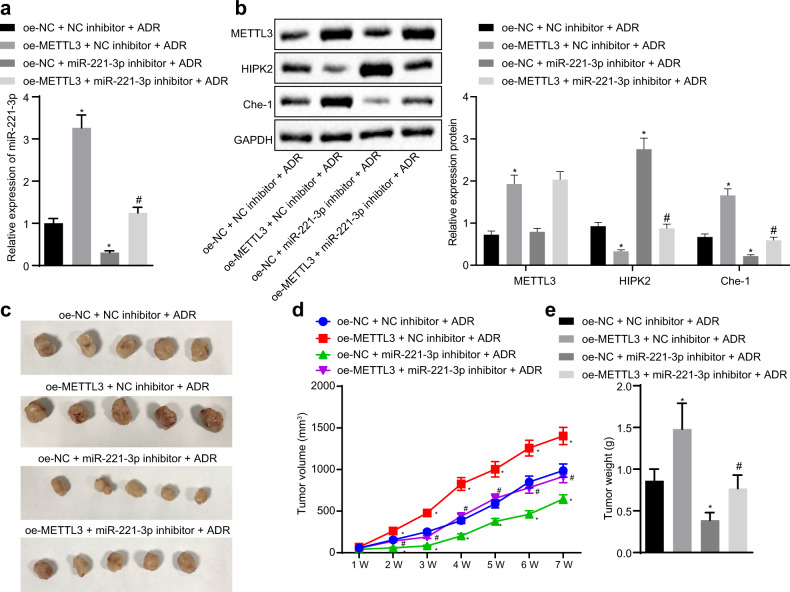
METTL3 promotes BC cell resistance to ADR by enhancing miR-221-3p expression in vivo. **a** The expression of miR-221-3p in mouse tumor tissues. **b** Immunoblots and quantification of HIPK2 and Che-1 in mouse tumor tissues, normalized to GAPDH. **c** Representative images of BC xenografts in nude mice. **d** The growth curve of BC xenografts in nude mice. **e** The weight of BC xenografts in nude mice. *p < 0.05 compared to oe-NC + NC inhibitor + ADR and #*p* < 0.05 compared to oe-NC + miR-221-3p inhibitor + ADR by Tukey’s test-corrected ANOVA or by Bonferroni-corrected repeated measures ANOVA for **d**.

To assess the impact of METTL3-mediated pri-miR-221-3p maturation on ADR resistance in vivo, we generated xenograft models of BC in nude mice by subcutaneously injecting MCF-7/ADR cells stably expressing METTL3 and/or miR-221-3p inhibitor. RT-qPCR and immunoblotting analysis (Fig. 6a, b) showed that METTL3 overexpression elevated the expression of miR-221-3p and Che-1 but diminished the expression of HIPK2 in mouse tumor tissues after ADR treatment. Inhibition of miR-221-3p by its specific inhibitor increased the expression of HIPK2 but reduced the expression of Che-1 in mouse tumor tissues after ADR treatment. In addition, METTL3 overexpression increased the growth of BC xenografts in nude mice, while miR-221-3p inhibition reduced the growth of BC xenografts in nude mice (Fig. 6c–e). Therefore, miR-221-3p inhibition was confirmed to negate the METTL3 overexpression-induced BC cell resistance to ADR in vivo.

## Discussion

In recent years, drug resistance has attracted increasing attention as a complex clinical condition involving various molecular alterations, highlighting a distinctive research direction to improve the survival of patients with BC^17,18^. In the present investigation, we explored the regulatory role and underlying mechanism of METTL3 in the drug resistance of BC cells. Collectively, the experimental data of our study demonstrated that silencing METTL3 reduced the drug resistance of BC cells to ADR, and promoted cancer cell apoptosis by downregulating miR-221-3p expression through inhibition of the m6A modification of pri-miR-221-3p.

A fundamental finding of our study showed a regulatory mechanism of METTL3 on miR-221-3p: METTL3 silencing induced by specific shRNA could reverse the expression pattern of miR-221-3p in BC cells by reducing m6A modification of pri-miR-221-3p. Consistently, the m6A methylase METTL3 has been revealed to be highly expressed in BC tissues and cells, while knockdown of METTL3 leads to accelerated apoptosis and suppressed proliferation of BC cells along with inhibited tumor growth^8^. Moreover, m6A modification of mRNAs has been implicated in multiple regulatory procedures at the post-transcriptional level and phenotypic progression of BC^19^. Largely in agreement with our finding, the oncogenic function of METTL3 has been validated in bladder cancer; METTL3 promotes the maturation of pri-miR-221/222 through mediation of m6A modification^7^. In addition, miR-221-3p has been reported to play a critical role in the histopathology and severity of BC^20^. High expression of miR-221 in BC tissues has been suggested to be closely associated with advanced clinical stage and poor oncologic outcomes of patients with BC^21^. Additionally, hsa-miR-221-3p has been found to be expressed at a high level when compared with its expression in matched tumor-free margin samples, functioning as a promising molecular biomarker for the diagnosis of BC^22^. Notably, miR-221 is commonly expressed with miR-222 in BC^23^. Additionally, both miR-221 and miR-222 can mediate tumor development through other signaling pathways and function as biomarkers for other diseases^24,25^. Therefore, future studies should be performed to explore the possible regulatory role of miR-222 in ADR-resistant cells, which may also be linked to the m6A methylase METTL3.

Furthermore, the following experiments demonstrated that the delivery of a specific inhibitor targeting miR-221-3p lead to reduced drug resistance of BC cells to ADR, suppressed cell viability and promoted cell apoptosis, as evidenced by diminished levels of MDR1, BCRP, and Bcl-2 along with elevated Bax levels. The association of the apoptosis-related factors Bcl-2 and Bax with BC tumorigenesis has been clarified; patients who are negative for distant metastasis with a lower degree of tumor differentiation exhibit lower Bcl-2 expression and higher Bax expression^26^. Accordingly, downregulated miR-221 induced by miR-221 inhibitor has been documented to inhibit the migratory and invasive properties of basal-like BC cells, reducing tumor aggressiveness^27^. Likewise, MDR1 knockdown has been found to reduce resistance of BC cells to cisplatin, contributing to chemotherapy sensitivity, while increased expression of MDR1 in peripheral blood of patients with solid breast tumors corresponds to metastasis and advanced tumor stage^28,29^. In addition, downregulation of BCRP has been shown to be indicative of enhanced chemosensitivity of BC cells^30^, supporting the results of our study.

Subsequent exploration of the downstream regulatory mechanism revealed that miR-221-3p resulted in chemoresistance of BC cells to ADR by mediating the expression of Che-1 by targeting and negatively regulating HIPK2. HIPK2, as an oncosuppressor related to the response to antitumor drugs, can be activated by ADR, and HIPK2 overexpression exerts inhibitory effects on BC cell invasion^31^. Recently, Che-1/AATF has emerged as a modulator of tumor cell survival and tumor progression, and silencing of AATF can be employed to trigger BC cell apoptosis^32,33^. HIPK2 has been identified as a proapoptotic kinase implicated in the degradation of Che-1, and the loss of HIPK2 results in reduction in Che-1 ubiquitylation and degradation^12^. Importantly, suppressed resistance to ADR is highly suggestive of impaired development and progression of BC^34–36^.

Taken together, our results provide novel mechanistic insights for a better understanding of the role of METTL3 in BC cell resistance to ADR via the functional axis of miR-221-3p/HIPK2/Che-1, offering a novel therapeutic strategy against the development and progression of BC. Ongoing and future prospective clinical studies are required to expand the validation of METTL3/miR-221-3p/HIPK2/Che-1 to enhance drug efficacy and reduce the side effects of chemotherapy in the management of BC. However, we recognize that additional mechanisms may be involved in the regulation of the reported pathway due to the complex microenvironment, which warrants further exploration.

## Supplementary information

Figure S1
